# Waiting for ASCO to “Walk the Walk”

**DOI:** 10.1093/oncolo/oyad256

**Published:** 2023-09-29

**Authors:** Tito Fojo

**Affiliations:** Department of Medicine, Division of Hematology Oncology, Columbia University, New York, NY, USA

## Abstract

This editorial addresses the magnitude of the challenge of spiraling drug costs, a challenge that affects everyone, everywhere, but may not be adequately addressed even at the ASCO annual meeting.

At the end of May, I and more than 40 000 of my closest friends, the overwhelming majority from higher income countries, met in Chicago at ASCO 2023 to hear the latest in oncology, much of it devoted to therapies that 95% of the world cannot or will soon not be able to afford. Al Neugut and Susan Bates took on the task of highlighting some of ASCO’s best offerings^1^—without contextualizing the highlights for drug cost. Here I will address four of the same abstracts plus one other example in which the magnitude of benefit and spiraling drug costs were not addressed in the ASCO presentations. These are problems that increasingly affect everyone, everywhere, and I will conclude that, unfortunately, on these critical issues ASCO still does not “walk the walk.”

## A Favorably Designed Study and the Real Problem With Cross-trial Comparisons

A glaring example emerged from the plenary presentation of the ADAURA trial, which evaluated osimertinib as adjuvant therapy for patients with stages IB-IIIA non-small cell lung cancer (NSCLC) harboring mutations in the epidermal growth factor receptor (EGFR).^2^ Placebo was given in the reference arm; the only thing more surprising than the choice of placebo was that the design received regulatory pre-approval. The rationale for choosing a placebo as the control—that none of the first- or second-generation EGFR tyrosine kinase inhibitors (EGFR TKIs) had garnered regulatory approval as adjuvant therapies and were, therefore, not regarded as standard of care (SOC)—is inadequate given the hundreds of trials that randomize against therapies lacking “regulatory approvals.” Three studies with both gefitinib and erlotinib as adjuvant therapies *in patients whose tumors harbored EGFR mutations* were ongoing at the time ADAURA was launched and all had standard chemotherapy—cisplatin plus vinorelbine—as the reference arms, not a placebo. ADJUVANT/CTONG1104 [NCT01405079] and EVAN [NCT01683175] showed significant improvements in disease-free survival (DFS) with erlotinib, achieving a significant improvement in overall survival (OS) (HR, 0.318; 95%CI, 0.151 to 0.670) in the phase II EVAN trial.^3-5^ The placebo control in ADAURA likely allowed osimertinib to achieve a far greater apparent magnitude of benefit than would have been observed with chemotherapy or a first generation TKI as a control.

In addition to selecting a placebo as the reference arm, osimertinib was given for three years whereas in the other ongoing studies, both gefitinib and erlotinib were being administered for only 2 years, an extra year of therapy that, as we learned with imatinib in gastrointestinal stromal tumors (GIST), could be very meaningful. Indeed, evidence that the duration of therapy is important can be found in ADAURA itself, in which the *rate* of disease recurrence increased 2.5-fold after the discontinuation of osimertinib at 36 months, suggesting that the value of EGFR TKIs in the adjuvant setting may be as treatment that can delay disease recurrence and, in turn, prolong survival rather than in bringing about cures.^6^ If this possibility is true, it means that the duration of therapy is a very important variable and it renders cross-trial comparisons difficult, leaving open the possibility that if administered longer, first generation EGFR TKIs could prolong OS.

But equally or more importantly, before its largest audience, ASCO ignored the elephant in the room—the staggering financial cost that will be incurred if osimertinib for adjuvant therapy is adopted worldwide as standard of care. In this context two important items were overlooked. The first was data from the APPLE trial [NCT02856893] that was presented earlier this year at the European Lung Cancer Congress^7,8^ in patients with metastatic disease in which comparable OS outcomes were achieved by leading with gefitinib and then turning to osimertinib only at the time of disease progression versus starting with osimertinib. Secondly, ASCO also ignored the potential of “me-too osimertinibs” to encourage competition; as one example, I mention aumolertinib, a drug that data suggest is as effective as osimertinib.^9^ The take home point should be that while we wait for aumolertinib and other cost-disrupting “osimertinibs” to come to market, enormous savings could be achieved by leading with gefitinib or erlotinib and continuing their administration for many years. It may be countered that osimertinib will reduce the emergence of brain metastases compared to gefitinib and erlotinib. But note that CNS recurrences occur with all EGFR-TKIs, including osimertinib. In AUDARA, with a median follow-up close to 4 years, CNS recurrences occurred in 6% of those assigned to osimertinib vs. 11% of placebo recipients. Critically missing from the comparison in the presentation were the percentages with gefitinib or erlotinib.

## The Only Thing Worse Than a Large Trial Is a Large Trial That Does Not Attempt to Identify the 2% to 3% That Benefit

Support for discouraging the design of trials that enroll thousands of participants was provided by NATALEE, a trial that added 3 years of adjuvant ribociclib to well-established, well-tolerated, and highly effective endocrine therapy in patients with HR+/HER2− breast cancer.^10^ Let us be clear about this—in the too often ignored 95% of the world, no one wants to add a drug with the marginal benefits that ribociclib achieved at 3 years—a 3.3% and 2.2% reduction in invasive disease-free survival (iDFS) and distant disease-free survival (DDFS), respectively, without an OS benefit. Quite frankly, few will want to do this in the well-heeled 5% of the world either, where an increasing number of patients are struggling with affording drugs with benefits this marginal. Further, ribociclib led to grade ≥ 3 liver-related AEs in 8.3% of patients and grade ≥ 3 neutropenia in an additional 43% of patients. Again, ignoring the elephant in the room, the exorbitant costs were not discussed, and the toxicities unscrutinized. Missing also was the never-conducted clinical trial that would have confirmed the fact that we all know - palbociclib, abemaciclib, and ribociclib are indistinguishable precluding speculation about the potential savings palbociclib, now available as a generic, might offer.

## Poorly Designing and Misinterpreting Non-inferiority Trials

Kudos to Italian investigators for conducting the definitive short-HER randomized phase III trial, which compared 9 weeks of trastuzumab to 1 year of trastuzumab for HER2-positive breast cancer in the adjuvant setting.^11^ The study was conducted with a non-inferiority design, in which the upper bound of the 90% confidence interval needed to be less than 1.3 but came in at 1.31.^11,12^ Two important considerations were overlooked. First, in a disease where the 10-year overall survival is 88%-89%, a 1.3 boundary can be seen as too narrow, even more so when enrolling a very heterogenous group of patients. Second, a 1.3 boundary may be considered proper for a trial of two comparable therapies where *efficacy is the only attribute being considered* as market share is pursued. However, if the therapy looking to be “non-inferior” has other benefits, such as fewer side effects, lower cost, or greater ease in delivery, a 1.31 boundary, may be acceptable, and still might be too strict.

The Short-HER regimen had all these positive attributes—significantly lower cardiotoxicity (HR 0.32, 95%CI, 0.21-0.50), lower costs (82% less trastuzumab!), and greater ease of delivery (9 weeks vs 1 year). These are attributes that are critically important everywhere, but especially in low- and middle-income countries, where short-HER should be immediately adopted. Notably, in an otherwise excellent presentation, less than ten seconds were dedicated to this key conclusion regarding the immense value of the Short-HER regimen. Instead, the speaker concluded “non-inferiority cannot be claimed and one-year of trastuzumab remains the standard of care.” This conclusion came right after noting that “based on a Bayesian analysis, the *probability* that 9 weeks are *not inferior* to 1-year trastuzumab is *93.2%*.” Which begs the question: At an annual meeting that routinely hails as a major success a “novel” therapy that is more toxic, more expensive and achieves more marginal benefits with *P*-values of 0.05 (95% probability), when did a 93.2% probability of “success” with a highly effective, less toxic, less expensive, and easier to administer intervention become not good enough?

## Pivoting to an Agent Where a Low-Cost Alternative Is More Likely to Emerge

A sliver of good news came in advanced stage Hodgkin lymphoma where we may have learned that when it comes to immunotherapy, less is likely the same as more and even the same as much more. Beginning in the 1980s and for more than 30 years, ABVD (Adriamycin, bleomycin, vinblastine, and dacarbazine) was the treatment of choice for Hodgkin lymphoma. Then, in 2018, investigators replaced bleomycin with brentuximab vedotin (SGN-35, Bv), an anti-CD30 antibody drug conjugate with a microtubule-targeting agent, monomethyl auristatin E (MMAE), as its payload and scored regulatory approvals at the expense of more neurotoxicity and G-CSF support.^13^ With Bv-AVD now the standard of care, a decision was made to remove Bv, replace it with nivolumab (N) and go head-to-head in 940 patients.^14^ The study presented at ASCO reported greater neutropenia with N-AVD, but only because less growth factor was used. Bv-AVD had more neuropathy, but N-AVD more immune AEs. One-year progression-free survival (PFS) was better with N-AVD, 94% to 88%. Although OS in both arms was very high, the trial had a median follow-up of only 12.1 months. The authors concluded “N-AVD is poised to be a new standard therapy for advanced stage cHL.”

A notable feature of this study was the 6 months of immunotherapy used in contrast to the 2-year duration widely adopted in other histologies. Ongoing trials in melanoma are examining shorter durations of immunotherapy, including 1 year for DANTE [ISRCTN15837212] and PET-Stop [NCT04462406], and intermittent versus continuous treatment in STOP-GAP [NCT02821013] or until confirmed CR/ongoing PR in Safe Stop [NCT05652673]. With eight nearly indistinguishable ICIs approved, we can stop hoping for competition to drive costs down, but the results in cHL are a good start on the road to more affordable durations of treatment. My bet—3 to 4 doses will eventually be shown to be sufficient. Meanwhile, ASCO should encourage the development of less expensive ICIs and shorter durations of treatment.

## Missing the Point as Results Are Exaggerated With a Regimen That Would Add Unnecessary Costs to an Accepted SOC

One final example is a poorly analyzed mesothelioma trial^15^ (not included in the Neugut-Bates highlights^1^). Worldwide, more than 35 000 new cases of mesothelioma are diagnosed each year with 30 000 deaths. Enter “CCTG IND.227: A Randomized Phase 3 Study of Chemotherapy versus Chemotherapy plus Pembrolizumab in Treatment-Naïve Pleural Mesothelioma” presented at ASCO 2023, providing solid evidence that few took ASCO seriously when it published a Perspective on “Raising the Bar for Clinical Trials by Defining Clinically Meaningful Outcomes” nearly a decade ago.^16^ This was a Perspective that never went anywhere despite its thoughtful conclusions and modest aspirational OS gains of 2.5 to 5 months to be achieved with novel therapies for diverse indications, ranging from first-line NSCLC to third- or fourth-line colorectal cancer. Fast forward to ASCO 2023 and triumphant claims that “endpoints were met” with a 1.15-month overall survival advantage by adding pembrolizumab to pemetrexed plus cisplatin for the therapy of mesothelioma, a claim that was astonishingly endorsed by the discussant.

More important was the missed opportunity to draw attention to the possible “true finding”—the potential value of adding pembrolizumab to cisplatin plus pemetrexed in the therapy of non-epithelioid mesothelioma. Comprising only 21.6% (95/440) of study participants, it was in those with a non-epithelioid histology, whose prognosis is worse, that benefit was potentially observed, an effect of a magnitude sufficient to “carry” the entire 440 patient cohort to statistical significance. Specifically, in the epithelioid cohort a hazard ratio of 0.89 (0.7-1.13) for OS clearly established that pembrolizumab is ineffective and should not be given alone nor added to cisplatin plus pemetrexed. But in the non-epithelioid cohort, a 4.1-month difference in OS with an HR of 0.57 (0.36-0.89) was very encouraging and worthy of ratification. I would note here these results emulate the data with nivolumab plus ipilimumab.^17^

In my view, concluding “platinum/pemetrexed and pembrolizumab is a new treatment option” in patients with previously untreated pleural mesothelioma based on a 1.15-month survival advantage that ignored the small fraction of patients with non-epithelioid histology with apparent actual benefit is indefensible because (i) a survival advantage of 1.15 months (35 days), even if real, falls woefully short of the modest aspirations of the ASCO Perspective and (ii) it ignored the ASCO mantra to identify those who may truly benefit from therapies that if administered to all can bring harm without benefit to the many.

## Conclusion

As a first step to walking the walk, it’s time for ASCO to publish an updated Perspective and make it required reading for all presenters, especially discussants. ASCO does many things very well, but if it is to walk the walk regarding affordability and deployment of drugs to more than 5% of the world, it must do more, much more, than occasionally have a session dealing with the challenges faced by low- and middle-income countries. Presentations must not just mention “financial toxicity,”^18,19^ but must deal with the true costs of “novel therapies.” Annual meetings badly lack and desperately need more critical assessment of trial shortcomings. Most importantly, honest assessments of the *true magnitude of benefits* and their often-associated toxicities must be presented while advocating not only for the “winner” but also for the now dethroned SOC that costs only a fraction but often delivers 95% or more of the value. Speakers must be encouraged to address these issues. Fortunately, oncologists are inherently optimistic. And so, we remain hopeful that someday as regards magnitude of benefit and drug costs, ASCO will walk the walk.
